# Perception of affordable diet is associated with pre-school children’s diet diversity in Addis Ababa, Ethiopia: the EAT Addis survey

**DOI:** 10.1186/s40795-024-00859-5

**Published:** 2024-03-06

**Authors:** Semira Abdelmenan, Hanna Y. Berhane, Christopher Turner, Alemayehu Worku, Katarina Selling, Eva-Charlotte Ekström, Yemane Berhane

**Affiliations:** 1 Global Health and Migration Unit, Department of Women’s and Children Health, Uppsala University, 751 85 Uppsala, Sweden; 2 Department of Epidemiology and Biostatistics, Addis Continental Institute of Public Health, Addis Ababa, Ethiopia; 3 Department of Nutrition and Behavioral sciences, Addis Continental Institute of Public Health, Addis Ababa, Ethiopia; 4 Institute of Public Health, College of Medicine and Health Sciences, University of Gondar, Gondar, Ethiopia; 5 Department of Population Health, Faculty of Epidemiology and Population Health, School of Hygiene and Tropical Medicine, London, UK

**Keywords:** Children, Dietary diversity, Affordability, Food environment, Nutrition, Ethiopia

## Abstract

**Background:**

Despite improvements in food access and nutrition security over the last few decades, malnutrition remains a major public health problem. One of the significant contributors to these problems is affordability of nutritious food. This study aimed to examine the association between perceived food affordability and pre-school children’s diet diversity in Addis Ababa, Ethiopia.

**Methods:**

Cross-sectional data from 2017 to 18 were used for the analysis. A 24-hour dietary recall assessment was done to assess children’s dietary diversity (DD). We used a modified operational definition of affordability indicator called perceived affordability of dietary diversity (afford-DD) to evaluate the impact of the food environment in terms of affordability at the household level. A sample (*n* 4,898) of children aged 6–59 months representative of households in Addis Ababa was randomly selected using a multistage sampling procedure including all districts in the city. Mixed-effects linear regression models were used to assess the association between children’s DD and afford-DD.

**Results:**

The survey revealed that the mean (standard deviation [SD]) of children’s DD was 3.9 [± 1.4] while the mean [SD] of afford-DD was 4.6 [± 2.1]. Overall, 59.8% of children met the minimum dietary diversity (≥ 4 food groups). White roots and tubers were the most commonly consumed food groups regardless of their affordability. Considerable variations were observed between households that reported the food item affordable and not affordable in consumption of Vitamin A rich vegetables and fruits, meat and fish, egg, and dairy. The children’s DD was positively associated with afford-DD after adjusting for maternal education, household wealth status and other relevant confounding. Higher maternal education modified the association between affordability and children’s diet diversity.

**Conclusions:**

This study suggests higher perceived food affordability was associated with better diet diversity in children. A higher level of maternal education had the potential to mitigate affordability challenges in meeting the children’s dietary diversity needs. Our study emphasizes the need for inclusive food programs and nutrition interventions addressing social differences, intensifying efforts to make nutrient-rich diets affordable for the less privileged, and highlights the potential benefits of targeting maternal education in addressing child dietary diversity.

**Supplementary Information:**

The online version contains supplementary material available at 10.1186/s40795-024-00859-5.

## Introduction

According to WHO 2021 report, undernutrition is estimated to be associated with 2.7 million child deaths annually, or 45% of all child deaths [1]. Infant and young child feeding is a key area to improve child survival and promote healthy growth and development. Globally in 2020, 149 million children under 5 were estimated to be stunted, 45 million were estimated to be wasted, and 38.9 million were overweight or obese [1]. Few children receive nutritionally adequate and safe complementary foods; in many countries, less than one-fourth of infants 6–23 months of age meet the criteria of dietary diversity and feeding frequency that are appropriate for their age [1].

Globally there are efforts to improve the dietary diversity and nutritional status of children by setting different targets including the sustainable development goals (SDGs): at least 12 of the 17 SDGs are directly or indirectly related to nutrition [2–4]. The World Health Assembly has a target for reducing stunting in children by 40% in 2025 [5]. Currently, the Ethiopian government also set targets to improve the nutritional status of children through the National Nutrition Program [6], and Health Sector Transformation Plan [7]. Ethiopia declared to end child malnutrition by 2030 [8, 9]. Despite the efforts, according to the 2016 demographic and health survey (DHS), 38% of children are stunted, 10% are wasted, and 24% are underweight [10].

The minimum dietary diversity score for children 6–23 months old is a population-level indicator designed by the WHO to assess diet diversity as part of infant and young child feeding (IYCF) practices among children. This indicator is one of the seven IYCF indicators developed by the WHO to provide simple, valid, and reliable metrics for assessing IYCF practices at the population level [11]. It is a measure of micronutrient adequacy of infant and young children’s diet [11]. In addition, minimum dietary diversity is used as a proxy indicator to assess the dietary diversity of older children aged 24–59 months assuming the older age group is worse off and can be an important alert indicator [12].

While both wealth of families [13] and maternal education [14, 15] have shown to be independently associated with children’s diet diversity (DD) less is known about the mechanisms of these factors. An obvious pathway of the wealth dimension is through increased affordability to buy food but there is limited research on food affordability. Further, it is reasonable to believe that maternal education in addition to having a direct effect on children’s DD, also can modify the association between food affordability and diet diversity.

There is a development in food environment measurements including price in the past few decades [16]. Affordability measurements consider the price of foods in either absolute, relative, or comparative terms. Various methods including objective and subjective approaches have been used to assess the affordability of food at the individual, household, or aggregate levels [16, 17]. Subjective methods include surveys of individual perceptions of affordability of food if the person or the family wants to consume the specific food items [18, 19]. Perception-based measures help detect variation in healthy food affordability and provide an in-depth understanding of individuals’ perceptions of all dimensions of the food environment [19, 20].

The present study uses a perception-based indicator of affordability of a healthy child’s diet to examine the effect of the affordability dimension of the food environment on child dietary diversity. The main objectives of this paper are (1) To examine the association between perceived food affordability and pre-school children’s DD; (2) To examine to what extent perception of food affordability affect children’s DD, and (3) To evaluate whether maternal education can modify the association between perceived food affordability and child dietary diversity.

## Methods

### Study setting and sampling

This study used cross-sectional data from the “What’s to eat? Women, children and the urban food environment: the case of Addis Ababa (EAT Addis study) in Ethiopia” study that has been described previously [21]. The survey was conducted in two rounds in 2017 and 2018 in Addis Ababa, the capital city of Ethiopia. Data were collected by trained interviewers using a tablet that contains an electronic version of the structured and piloted questionnaire designed in Open Data Kit [22].

The study followed a multi-stage sampling procedure. In the first stage, each woreda in the city of Addis Ababa was divided geographically into five equal clusters, of which one was selected randomly. In the second stage, 60 households from each cluster were selected using a systematic sampling procedure. A total of 14,018 households were visited to identify eligible households and 4,898 households were included in the current analysis (supplemental file Fig. 1). All household with at least one under-five child was eligible to participate. If several children under 5 years were present, one child was randomly selected for the dietary recall. In this study, all children aged six months to 59 months were included in the analysis.

**Fig. 1:**
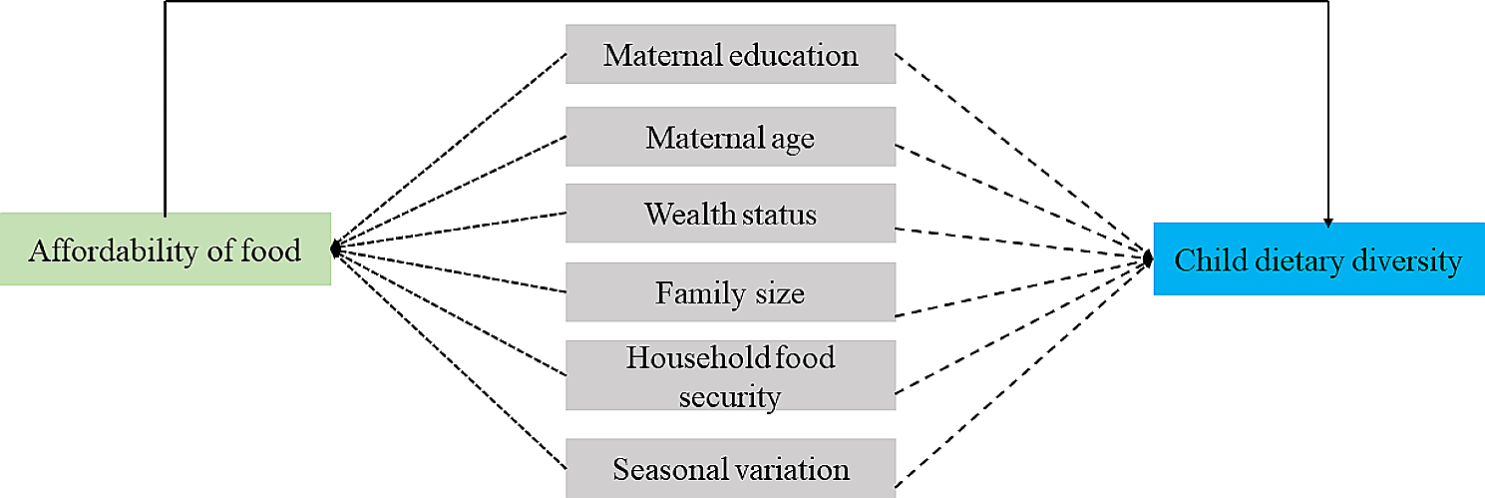
Hypothesized relationship between child dietary diversity and food affordability. The main exposure variable is shown in green, the outcome variable is shown in blue, and potential confounders from prior research are shown in grey. An arrow from a factor to another means possible associations. Broken arrows indicate confounders to adjust for, while bold arrows represent the current analysis

### Outcome variables

Child food consumption was assessed using a 24-hour recall method. Mothers were asked to recall the child’s meals, including beverages and snacks, consumed in the preceding 24 h. To ensure a comprehensive list of food items, we employed a two-step approach. Initially, mothers were questioned about the consumed food items from their memory. Subsequently, to enhance the completeness of the list, mothers were asked to recall the ingredients used in the preparation of each meal mentioned. To facilitate the recall process, a photo-based tool was utilized. This tool featured images of the most common food items organized according to their respective food groups. Mothers were encouraged to refer to these visual aids to provide a more accurate account of the foods consumed by the child. Based on the WHO guidelines [11] for infant and young children feeding practices, the food lists were grouped into seven food groups: (1) cereals, white roots, and tubers, (2) legumes, nuts, and seeds, (3) dairy products, (4) meat and fish, (5) eggs, (6) vitamin A-rich vegetables and fruits, and (7) other fruits and vegetables. The children’s DD was calculated by adding the number of food groups out of seven and further categorized into adequate (4 or more groups) or inadequate (< 4) diet diversity.

### Key independent variables

#### Perceived affordability of a diverse diet for children

Perceived affordability of food was asked for each food items considered as recommended nutritious food based on the WHO guidelines [23]for infant and young children feeding practices. Food affordability was assessed by asking the mothers “How often can your family afford to consume any of these foods?”. The response options were “as often as wanted”, “a little less frequently than wanted”, and “much less frequently than wanted/not at all”. For our analyses, we defined ‘perceived affordable’ when the response was as often as wanted and ‘perceived not affordable’ for the other responses. The affordable dietary diversity (afford-DD) was calculated using the score of affordable food groups out of the seven recommended food items included in children’s dietary diversity. Similarly, the cut-off points of at least 4 of the 7 food groups were used to further dichotomized afford- DD into adequate and inadequate.

##### Maternal education

The level of education was assessed by asking the mother to indicate their highest education level. Maternal education was categorized into five groups: “Never attend school/ Not finished first grade”, “Grade 1–4”, “Grade 5–8”, “Grade 9–12”, and “college”.

##### Wealth quintiles

The wealth index was computed as a measure of family wealth using principal component analysis. The variables included in the measure were ownership of a house, household assets, materials used for housing construction, ownership of water source, and sanitation facility. The generated principal component was divided into five equal quintiles: lowest, second, middle, fourth, and highest.

### Statistical analysis

Descriptive analyses were conducted to examine the sociodemographic and diet-related characteristics of the participating children. Continuous variables: child dietary diversity and affordability of dietary diversity were summarized using mean and standard deviations. Categorical variables: maternal age, maternal education, wealth quintiles, food security, and child age, were summarized using frequency and percentages.

We used a conceptual diagram to represent the hypothesized pathways from affordability of food to child dietary diversity based on evidence from the conceptual framework to maternal and child nutrition developed by the United Nations Children’s Fund (UNICEF) in 1990 [24] and further updated in 2020 [25] and the food environment conceptual framework developed by different researchers [16, 20, 26]. In Fig. 1 we present the directed acyclic graph (DAG) [27] that was used to determine the confounders that were introduced into the statistical models. Multicollinearity among affordability of food, maternal education and wealth status were assessed using variance inflation factors (VIF). The mean value of the VIF was 1.08 which indicated there was no multicollinearity.

Pearson correlation coefficient (r) was used to examine the correlation between children’s DD and afford-DD. Mixed-effects linear regression models accounted for the clustering in data were used to assess the association between the independent and outcome variables. The first model assessed children’s DD with each of the key independent variables: affordability of dietary diversity, maternal education, and wealth quintiles. The second model was adjusted for affordability of dietary diversity, maternal education, and wealth quintiles. The third model included additional confounders: maternal age, family size, household food insecurity and season. A p*-value* of < 0.05 was considered statistically significant. Maternal education was tested as an interaction term with child dietary diversity scores. All analyses were conducted using Stata version 16.0 statistical software [28].

## Result

### Characteristics of the study participants

A total of 4,898 participants were included in the analysis. The sociodemographic and household characteristics of the study participants are shown in Table 1. More than one-fourth of the mother who participated in the study were under 35 years of age, the majority of them were currently married (87.5%), 59.5% of the children were in the age group 24–59 months, the mean family size was 4.3 [± 1.5 SD], the mean of child dietary diversity was 3.9 [± 1.4 SD] while the mean affordable dietary diversity was 4.6 [± 2.1SD].

**Table 1:** Demographic information of the participating households in the EAT Addis study, Addis Ababa, Ethiopia

			Total
Level	Variables (*N* = 4,898)		n(%)/mean [SD]
Child	Sex	Male	2,561 (52.3%)
		Female	2,337 (47.7%)
	Age	6–23	1,986 (40.5%)
		24–59	2,912 (59.5%)
	Diet diversity		3.9 [1.4]
	Affordable diet diversity		4.6 [2.1]
Maternal	Age	15–24	736 (15.0%)
		25–34	2,990 (61.0%)
		35–44	925 (18.9%)
		45 and above	247 (5.0%)
	Education	Never attend/Not finished first grade	693 (14.1%)
		Grade 1–4	442 (9.0%)
		Grade 5–8	1,462 (29.8%)
		Grade 9–12	1,351 (27.6%)
		College	950 (19.4%)
	Marital status	Currently married	4,285 (87.5%)
		Currently unmarried	613 (12.5%)
Household	Food security	Food secure	2,997 (61.2%)
		Mildly food insecure	456 (9.3%)
		Moderately food insecure	958 (19.6%)
		Severely food insecure	487 (9.9%)
	Household size		4.3 [1.5]
	Season	Wet	2,512 (51.3%)
		Dry	2,386 (48.7%)
	Wealth	Lowest	978 (20.0%)
		Second	973 (19.8%)
		Middle	979 (20.0%)
		Fourth	984 (20.1%)
		Highest	984 (20.1%)

Data are presented as mean [SD] for continuous measures, and % (n) for categorical measure

### Child food consumption

Our further analyses by affording the food group revealed that cereals, white roots, and tubers were the most commonly consumed food groups regardless of their affordability; 91.2% of households reported food items affordable and 88.4% of households reported the food items not affordable. Other food groups most commonly consumed in all households are other fruits and vegetables (90.9% of households reported affordable vs. 80.6% of households reported not affordable) and legumes/nuts/seeds (75.2% of households reported affordable vs. 66.8% of households reported not affordable). Considerable variations were observed between households that reported the food item affordable and not affordable in consumption of Vitamin A-rich vegetables and fruits (42.5% vs. 19.3%), meat and fish (42.5% vs. 16.8%), egg (37.8% vs. 13.8%), and dairy products (69.6% vs. 21.1%). The largest variation was observed for dairy products, with a ratio of 3.3, indicating that households reporting affordability were 3.3 times more likely to consume dairy products compared to those reporting non-affordability. Similarly, the ratios for eggs (2.7 times) and meat and fish (2.5 times) highlight substantial differences in consumption patterns between the affordable and non-affordable food groups. Overall, Table 2 shows that there is low consumption of micronutrient-rich and animal-source protein-rich foods.

**Table 2:** Child food consumption by the affordability of the food group in the EAT Addis study, Addis Ababa, Ethiopia

Food groups	Food consumption by affording the food group				
	Yes		No		
	n(N)	%(95%CI)	n(N)	%(95%CI)	Ratio
Cereal/w root/tub	3984/4336	91.2 (90.4, 92.1)	471/532	88.4 (85.4, 90.8)	1.0
LNS	3104/4130	75.5 (73.8, 76.5)	514/768	66.8 (63.4,70.0)	1.1
Dairy products	1718/2468	69.6 (67.8,71.8)	511/2430	21.1 (19.5,22.7)	3.3
Meat and fish	720/1692	42.5 (40.2, 44.9)	540/3206	16.8 (15.6,18.5)	2.5
Eggs	920/2430	37.9 (36.0,39.8)	339/2468	13.9 (12.6,15.3)	2.7
Vit A vegs and fruits	1457/3429	42.5 (40.8, 44.1)	283/1469	19.3 (17.4, 21.4)	2.2
Other vegs and fruits	3868/4253	90.9 (90.0, 91.7)	520/645	80.6 (77.3, 83.4)	1.1

‘Yes’ and ‘No’: Indicate households reporting the respective food group as affordable and not affordable, respectively

n(N): The ‘n’ value represents the number of households within each specific food group, and ‘N’ represents the total number of households

Percentage (%): Represents the prevalence of food consumption within each affordability category along with the 95% confidence interval

Ratio: Indicates the ratio of the prevalence between ‘Yes’ and ‘No’ categories, offering insight into the differences in consumption patterns based on affordability

Abbreviations: Cereal/w root/tub- Cereals, white roots and tubers; LNS-Legumes, nuts and seeds; Vit A veg and fruits- Vitamin A rich vegetables and fruits; Other vegs and fruits- Other vegetables and fruits

Table 3 presents an analysis of children’s dietary diversity based on affordability, maternal education, and wealth quintile. The unadjusted means and proportions for diet diversity and the proportion meeting the minimum dietary diversity threshold (≥ 4 food groups) are reported for different categories within each variable. Statistical significance is indicated by p-values, assessing differences between groups. The survey revealed that overall, 59.8% (CI: 58.4, 61.2) of children met the minimum dietary diversity (≥ 4 food groups). The subsequent analysis presented in Table 3 shows the dietary diversity of children by affordability, wealth, and maternal education. Notably, children from households with adequate affordability of diverse diets (69.2%) showed a significantly higher proportion of meeting the minimum dietary diversity compared to those from households with inadequate affordability (37.2%). Children from college-level educated mothers had significantly higher DD (4.4) compared to children from never educated mothers (3.4). The proportion of children meeting the minimum DD was significantly higher in children with college-level educated mothers (75%) compared to children from never educated mothers (42.4%). However, no significant difference was observed in the two lowest educational levels. The wealth quantile showed similar variation as maternal education where children from the highest wealth quintile households had significantly higher DD than children from the poorest wealth quantile. The proportion of children meeting the minimum DD was significantly higher in children from the highest wealth quintile households (76.1%) than compared to children from the poorest wealth quintile (43.1%).

**Table 3:** Dietary diversity of children by affordability, wealth, and maternal education in the EAT Addis study, Addis Ababa, Ethiopia

Variable *(n* *=* *4898)*	Affordability						
	Inadequate afford-DD			Adequate afford-DD			p-Value
Diet diversity	3.3 [± 1.3]			4.1 [± 1.4]			< 0.001
Adequate DD	37.2 (34.7, 39.7)			69.2 (67.7,70.7)			< 0.001
**Education**							
	**Never attend**	**Grade 1–4**	**Grade 5–8**		**Grade 9–12**	**College**	***p-Value***
Diet diversity	3.4 [± 1.2]	3.4 [± 1.3]	3.7 [± 1.4]		4.1 [± 1.4]	4.4 [± 1.5]	< 0.001
Adequate DD	42.2 (38.8, 46.1)	43.4 (38.9, 48.1)	56.0 (53.5, 58.6)		67.3 (64.7, 69.7)	75.4 (72.5,78.0)	< 0.001
**Wealth**							
	**lowest**	**second**	**middle**		**fourth**	**highest**	***p-Value***
Diet diversity	3.4 [± 1.3]	3.7 [± 1.3]	3.9 [± 1.4]		3.9 [± 1.5]	4.4 [± 1.5]	< 0.001
Adequate DD	43.1 (40.0, 46.2)	54.5 (51.3, 57.6)	62.5 (59.4, 65.5)		62.9 (59.8, 65.9)	76.1 (73.3,78.6)	< 0.001

Values are presented as mean [SD] for continuous measures, and % (95% CI) for categorical measures

Adequate afford-DD indicates that the family can afford to consume at least four out of the seven WHO-recommended nutritious food items “as often as wanted”

Inadequate afford-DD indicates a score below four out of the seven WHO-recommended nutritious food items

### Does food affordability affect the children’s diet?

**Fig. 2:**
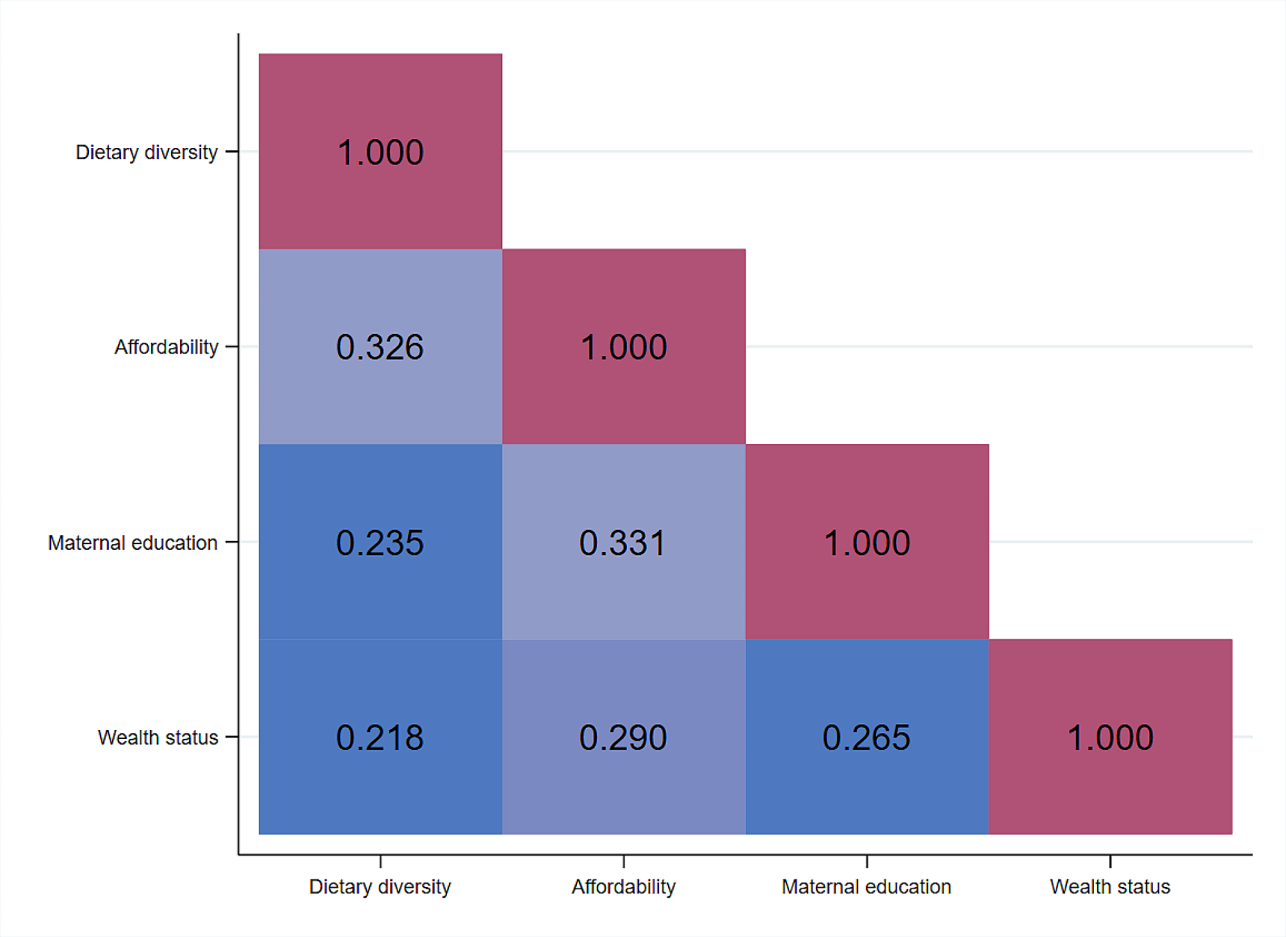
Correlation matrix of child dietary diversity, affordability of food, maternal education and wealth status with Pearson’s correlation coefficients (r). All correlations were significant at *p* < 0.001

The correlation of child dietary diversity score with key factors analysis showed that child dietary diversity had a positive correlation to affordability of food (*r* = 0.33). An increase in maternal education positively correlated with child DD score (*r* = 0.24). Furthermore, wealth status has a positive correlation with Child DD score (*r* = 0.22). All correlations were significant at *p* < 0.001 (Fig. 2).

**Fig. 3:**
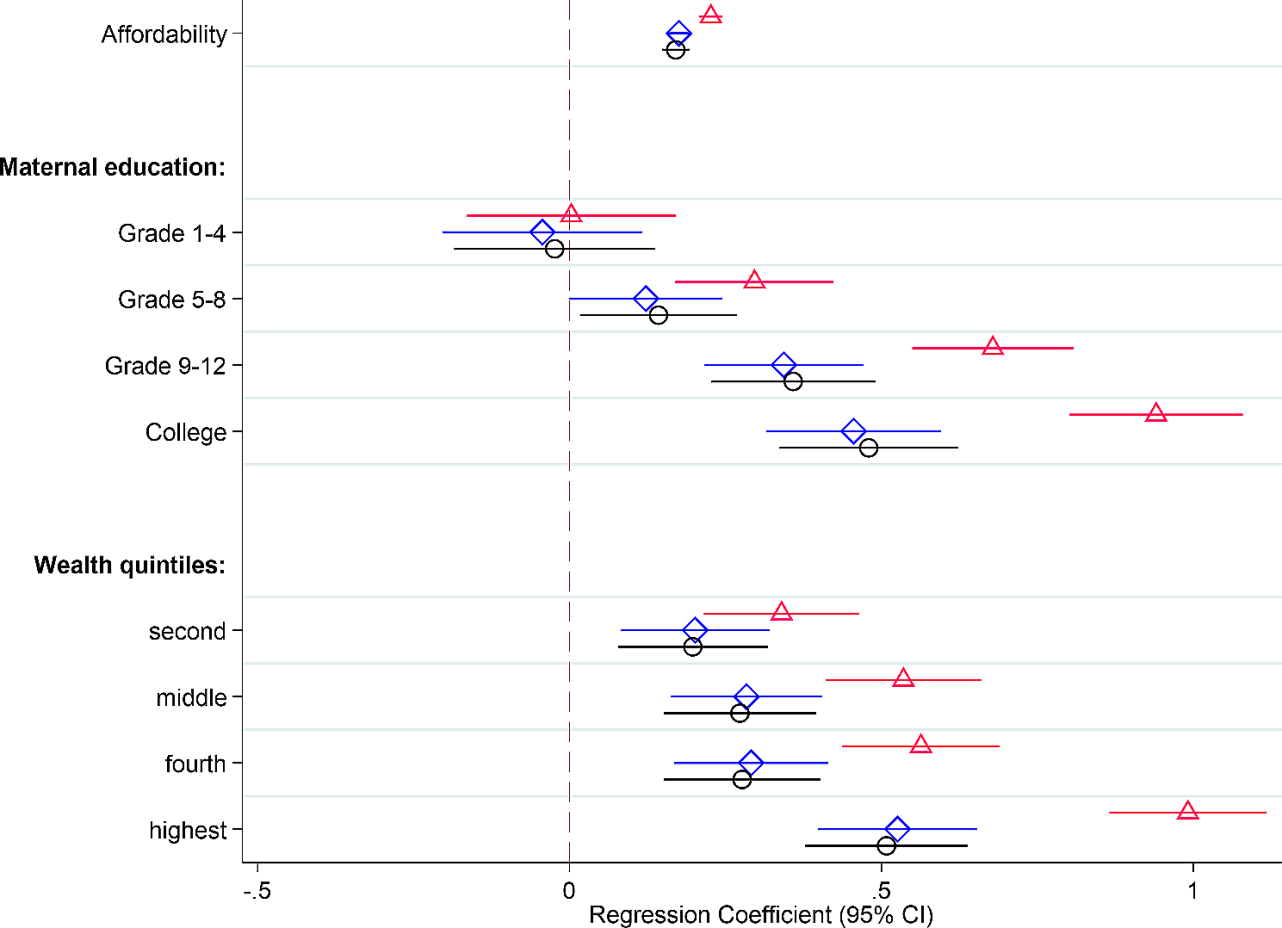
Parameter estimates and 95% confidence intervals (CI) for the association between afford-DD on the outcome child dietary diversity of unadjusted and adjusted models accounted for the clustering in data. Model 1: bivariate models (△); Model 2: multivariable model (◇) adjusted for affordability of food, maternal education and wealth status; Model 3: multivariable model (O) adjusted for affordability of food, maternal education, wealth status, maternal age, family size, seasonal variation, and household food insecurity

The results of multivariable mixed effect linear regression models showed that affordability of food was positively associated with the children’s dietary diversity. After controlling for maternal education, and wealth quintiles, a one-unit increase in affordability score resulted in an increase of 0.18 diet diversity scores (95% CI: 0.16,0.20). One unit increase in affordability of food was associated with increased children’s dietary diversity (0.17, 95% CI: 0.15,0.19), even after adjusting for maternal age, family size, seasonality, and household food insecurity in addition to maternal education and household wealth status (Fig. 3 and supplemental Table 1). Children from college-level educated mothers had higher DD (0.46, 95% CI: 0.32,0.60) compared to children from mothers with no education. The same difference in children’s DD scores was found between children from households with the highest and lowest wealth (0.53, 95% CI: 0.40,0.65).

### Does maternal education modify the effect of affordability of dietary diversity on child dietary diversity scores?

**Fig. 4:**
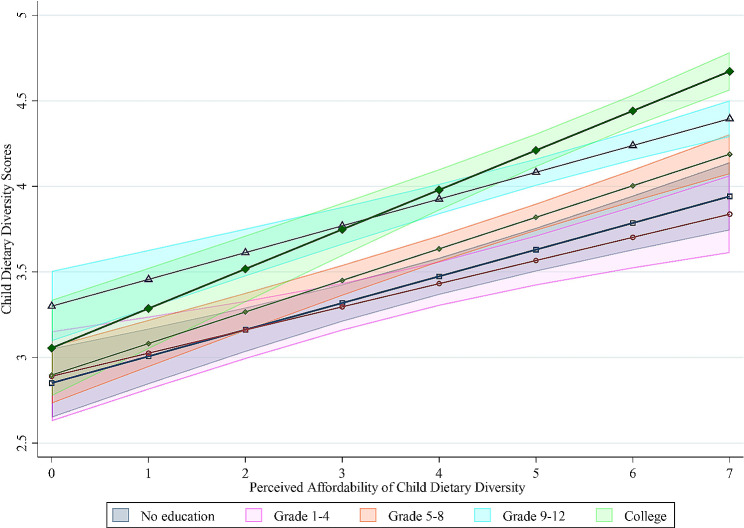
Visualization of the two-way interaction between afford-DD and maternal educational level on the outcome child dietary diversity. The results are derived by fitting a mixed-effect linear regression model to the data that accounts for data clustering, and by including afford-DD and maternal educational levels as fixed predictors, including their interaction

Figure 4 shows the interaction between afford-DD and the level of maternal education concerning child-DD. We found a positive relationship between afford-DD and Child-DD in Addis Ababa, suggesting that perceived affordability is strongly and positively associated with better child-DD for children with college-level educated mothers. Among mothers who attended college-level education, affordability of dietary diversity was associated with higher child dietary diversity scores (0.08, 95% CI: 0.01,0.14). However, there was no significant interaction observed between mothers with no education, primary, or secondary level education, and child dietary diversity.

## Discussion

In a sample of 4,898 participants in Addis Ababa, Ethiopia, we described the diet of children aged 6 to 59 months. In addition, the current study assessed whether the perceived affordability of dietary diversity is associated with child dietary diversity among children in an urban area. We used a modified operational definition of affordability indicator called perceived affordability of dietary diversity to evaluate the impact of the food environment in terms of affordability at the household level.

. Almost half (40.2%) of the children had inadequate dietary diversity, that is, less than 4 types of food categories in their regular diet. This finding is similar to the study conducted in Addis Ababa [29] and southern Ethiopia [30], nevertheless, it is higher than the reported national 2019 mini-EDHS [31] and a systematic review and meta-analysis conducted in Ethiopia [32]. The variation might be related to the sample size, study area, and time of the study. Even though this study and the above-mentioned studies used a cross-sectional method, EDHS is a community based national representative study that was conducted in rural communities and the meta-analysis included studies mostly conducted in a rural community while our study was conducted in an urban area. In addition, our study included a large sample size ensuring representativeness of the resident of the city while the other focuses on national representativeness or the specifically selected community.

The perceived affordability of DD was higher (4.6 [± 2.1]) compared to children DD. Regardless of the affordability the following food groups: cereals, white roots and tubers, legumes, and other vegetables are commonly consumed. These results are not unexpected since the mentioned food groups are the main items of staple food in Addis Ababa. They are consumed for survival even if the family perceived them as not affordable. Consumption of vitamin A-rich vegetables and fruits, meat and fish, dairy, and eggs showed a significant variation based on affordability. This result shows that while there are many factors affecting the consumption of the recommended nutrient-rich diversified foods, affordability of food remains among the important one. Our findings are consistent with other studies [33, 34], even though they have used different methods to assess affordability, suggesting that affordability impacts the diet diversity of children.

Our analysis also reveals that irrespective of maternal education, and wealth status, children from households who reported to afford adequate DD were more likely to consume a diverse diet than those who reported not to afford for the minimum recommendation of DD. This is supported by the global analysis of affordability and diet [35, 36], however, the mentioned studies used income and retail price to estimate affordability. Diets with adequate nutrients are unaffordable for a different group of the population and improving the affordability of diet, especially in low- and middle-income countries requires improvement in income and adjustment in the price of food.

The results of our study suggest a positive association between afford-DD and children’s dietary diversity, a finding aligned with other studies [21, 29, 34]. The possible reason could be despite awareness and knowledge of the dietary recommendation, the personal food environment such as the perceived affordability are still barriers to feeding children a diversified food. Qualitative studies conducted in Addis Ababa to assess factors influencing the decisions on what to feed pre-school children [37] and adolescents’ dietary behaviors [38] concluded that concerns related to affordability are among the dominating influencing factors of food choices. Unfortunately, the low- and middle-income countries’ external food environment in terms of price is characterized as highly unstable price, vulnerable to shocks and seasonality, relatively unhealthy and quick-fix foods are cheap [39], which makes the problem worse.

In our study, there were interaction effects between afford-DD and level of maternal education on children’s dietary diversity. There are several possible explanations for these observed interactions. The association between maternal education and child-DD is well established. Higher-level maternal educational attainments were significantly associated with higher dietary diversity [14, 21, 29, 31, 40] compared to those who do not have formal education. This could be because educated mothers have a better understanding of the importance of diversified food, and better knowledge of child feeding practices with easily available and affordable food items. The statistically significant interaction between afford-DD and level of maternal education demonstrates the moderating effect that level of education can have on the affordability of diversified foods.

This study has several strengths. The EAT Addis study population includes all administrative units of the city of Addis Ababa that provided a wide range of representativeness of the community. The study has a large sample size that covers a considerable age group and both genders. The response rate was high with the major respondent being the mother, which is expected to provide a high accuracy response. Most of the existing studies on child dietary diversity focused on other individual and households level factors. Our study fills the gap by providing evidence on the role of affordability on child dietary diversity.

It is important to note the limitations of our study. The study establishes a possible association between child dietary diversity and perceived affordability of child dietary diversity, not causality. In addition, perceived affordability of dietary diversity and food consumption can both be liable to social desirability bias. However, we tried to minimize social desirability bias by clearly explaining the study objectives and neutrally asking the questions. Therefore, although it is impossible to avoid the possible bias altogether, the impact is likely minimal due to the measures we have taken.

In conclusion, we found that child dietary diversity is associated with perceived affordability of dietary diversity after adjusting for maternal education, household wealth status and other relevant variables from existing literatures. Our result also showed that higher level maternal education has the potential to mitigate the effects of perceived affordability of child dietary diversity on children’s dietary diversity. Thus, food programs and nutrition interventions need to account for social differences to improve dietary diversity of children and efforts need to be intensified to make healthy diets with adequate nutrients affordable to the less privileged segments of society, including improving income and making food prices affordable. This finding indicates that programs targeting maternal education may be beneficial in addressing the issue of child dietary diversity. Furthermore, interventions should consider implementing health education initiatives that raise awareness of the importance of eating a healthy and strategies to diversify one’s diet using affordable food items. Additionally, more research is needed to examine the role of affordability and its effect on children’s dietary diversity in low- and middle-income countries.

### Electronic supplementary material

Below is the link to the electronic supplementary material.

Supplementary Material 1

## Data Availability

Data described in the manuscript, code book, and analytic code are available from the corresponding author and provided for a reasonable request.

## Abbreviations

afford-DD: Affordability of Dietary Diversity
CI: Confidence intervals
DAG: Directed Acyclic Graph
DHS: Demographic and Health Survey
DD: Dietary diversity
IYCF: Infant and Young Child Feeding
SDGs: Sustainable Development Goals
UNICEF: United Nations Children’s Fund
VIF: Variance inflation factors
WHO: World Health Organization

## Declarations

### Authors’ information

^1^ Global Health and Migration Unit, Department of Women’s and Children Health, Uppsala University, Uppsala, Sweden.^2^Department of Epidemiology and Biostatistics, Addis Continental Institute of Public Health, Addis Ababa, Ethiopia. ^2^Department of Nutrition and Behavioral sciences, Addis Continental Institute of Public Health, Addis Ababa, Ethiopia.^4^Institute of Public Health, College of Medicine and Health Sciences, University of Gondar, Gondar, Ethiopia.^5^Department of Population Health, Faculty of Epidemiology and Population Health, London School of Hygiene and Tropical Medicine, London, UK.

## References

[1] World Health Organization. Infant and young child feeding [Internet]. [cited 2021 Nov 24]. Available from: https://www.who.int/news-room/fact-sheets/detail/infant-and-young-child-feeding.

[2] Robert KW, Parris TM, Leiserowitz AA (2005). What is Sustainable Development? Goals, indicators, values, and practice. Environ Sci Policy Sustain Dev.

[3] Griggs D, Stafford-Smith M, Gaffney O, Rockström J, Öhman MC, Shyamsundar P (2013). Sustainable development goals for people and planet. Nature.

[4] GBD 2015 SDG Collaborators (2016). Measuring the health-related Sustainable Development Goals in 188 countries: a baseline analysis from the global burden of Disease Study 2015. Lancet Lond Engl.

[5] World Health Organization (2014). Global Nutrition targets 2025: stunting policy brief.

[6] MOH, Ethiopia. National nutrition program 2016–2020 - Google Scholar [Internet]. [cited 2021 Nov 24]. Available from: https://scholar-google-com.ezproxy.its.uu.se/scholar_lookup?title=National+Nutrition+Program+2016%E2%80%932020&publication_year=2015&amp.

[7] MOH. Federal Democratic Republic of Ethiopia Ministry.; Health sector transformation plan [Internet]. [cited 2021 Nov 24]. Available from: https://scholar-google-com.ezproxy.its.uu.se/scholar_lookup?title=Ministry+of+Health.+Health+Sector+Transformation+Plan+(2015/16%E2%80%932019/20)&publication_year=2015&amp.

[8] MOH, Brief. Reducing Stunting in Ethiopia:From Promise to impact [Internet]. [cited 2021 Nov 24]. Available from: https://scholar-google-com.ezproxy.its.uu.se/scholar_lookup?title=An+Evidence+Informed+Policy+Brief+Reducing+Stunting+in+Ethiopia:+%E2%80%9CFrom+Promise+to+Impact%E2%80%9D&publication_year=2019&

[9] MOH. Seqota Declaration Implementation Plan. (2016–2030): Summary Program Approach [Internet]. [cited 2021 Nov 24]. Available from: https://scholar-google-com.ezproxy.its.uu.se/scholar_lookup?title=Seqota+Declaration+Implementation+Plan+(2016%E2%80%932030):+Summary+Program+Approach+Document&publication_year=2016&amp.

[10] CSA/Ethiopia CSA, Ethiopia Demographic ICF, Health Survey. 2016. 2017 Jul 1 [cited 2021 Nov 24]; Available from: https://dhsprogram.com/publications/publication-FR328-DHS-Final-Reports.cfm.

[11] World Health Organization. Indicators for Assessing Infant and Young Child Feeding Practices: Part 1: Definitions: Conclusions of a Consensus Meeting Held 6–8 November 2007 World Health Organization. Washington, DC, USA; 2008.

[12] Minimum dietary diversity for. children 24–59 months| Indicator Registry [Internet]. [cited 2021 Dec 4]. Available from: https://ir.hpc.tools/applications/ir/indicator/n-018.

[13] Eshete T, Kumera G, Bazezew Y, Mihretie A, Marie T (2018). Determinants of inadequate minimum dietary diversity among children aged 6–23 months in Ethiopia: secondary data analysis from Ethiopian Demographic and Health Survey 2016. Agric Food Secur.

[14] Fentaw Mulaw G, Wassie Feleke F, Adane Masresha S (2020). Maternal Characteristics Are Associated with Child Dietary Diversity score, in Golina District, Northeast Ethiopia: A Community-based cross-sectional study. J Nutr Metab.

[15] University BY, Provo UT, Crookston USA, Bennett BT, Hall C, Hasan PC, Linehan M, Department of Public Health (2018). Increased Maternal Education and Knowledge of Nutrition and reductions in Poverty are Associated with Dietary Diversity and Meal frequency in an observational study of Indonesian children. Int J Child Health Nutr.

[16] Herforth A, Ahmed S (2015). The food environment, its effects on dietary consumption, and potential for measurement within agriculture-nutrition interventions. Food Secur.

[17] Charreire H, Casey R, Salze P, Simon C, Chaix B, Banos A (2010). Measuring the food environment using geographical information systems: a methodological review. Public Health Nutr.

[18] Leroy JL, Ruel M, Frongillo EA, Harris J, Ballard TJ (2015). Measuring the Food Access Dimension of Food Security: a critical review and mapping of indicators. Food Nutr Bull.

[19] Moore LV, Diez Roux AV, Brines S (2008). Comparing perception-based and Geographic Information System (GIS)-Based characterizations of the Local Food Environment. J Urban Health.

[20] Turner C, Kadiyala S, Aggarwal A, Coates J, Drewnowski A, Hawkes C et al. Concepts and methods for food environment research in low and middle income countries. Agric Nutr Health Acad Food Environ Work Group ANH-FEWG Innov Methods Metr Agric Nutr actions IMMANA Programme Lond UK. 2017.

[21] Abdelmenan S, Berhane HY, Jirström M, Trenholm J, Worku A, Ekström EC (2020). The Social Stratification of Availability, affordability, and Consumption of Food in Families with preschoolers in Addis Ababa; the EAT Addis Study in Ethiopia. Nutrients.

[22] Hartung C, Lerer A, Anokwa Y, Tseng C, Brunette W, Borriello G. Open data kit: tools to build information services for developing regions. In: Proceedings of the 4th ACM/IEEE International Conference on Information and Communication Technologies and Development [Internet]. New York, NY, USA: Association for Computing Machinery; 2010 [cited 2022 Apr 8]. p. 1–12. (ICTD ’10). 10.1145/2369220.2369236.

[23] World Health Organization (2021). Indicators for assessing infant and young child feeding practices: definitions and measurement methods.

[24] UNICEF. Strategy for improved nutrition of children and women in developing countries. 1990 [cited 2022 Jul 11]; Available from: https://digitallibrary.un.org/record/132779.

[25] UNICEF. UNICEF Conceptual Framework on maternal and child nutrition [Internet]. 2021 [cited 2022 Jun 6]. Available from: https://www.unicef.org/documents/conceptual-framework-nutrition.

[26] Drewnowski A, Finley J, Hess JM, Ingram J, Miller G, Peters C (2020). Toward healthy diets from sustainable Food systems. Curr Dev Nutr.

[27] Textor J, van der Zander B, Gilthorpe MS, Liśkiewicz M, Ellison GT (2016). Robust causal inference using directed acyclic graphs: the R package ‘dagitty’. Int J Epidemiol.

[28] StataCorp (2019). Stata Statistical Software: release 16. College Station.

[29] Solomon D, Aderaw Z, Tegegne TK (2017). Minimum dietary diversity and associated factors among children aged 6–23 months in Addis Ababa, Ethiopia. Int J Equity Health.

[30] Sagaro G, Alemayehu M (2017). Dietary diversity and Associated factors among infants and Young Children in Wolaita Zone, Southern Ethiopia. Sci J Clin Med.

[31] Ethiopian Public Health Institute (EPHI), ICF. Ethiopia Mini Demographic and Health Survey 2019: Final Report [Internet]. Rockville, Maryland, USA: EPHI and ICF; 2021 [cited 2022 Apr 11]. Available from: https://dhsprogram.com/pubs/pdf/FR363/FR363.pdf.

[32] Temesgen H, Negesse A, Woyraw W, Mekonnen N (2018). Dietary diversity feeding practice and its associated factors among children age 6–23 months in Ethiopia from 2011 up to 2018: a systematic review and meta-analysis. Ital J Pediatr.

[33] IFPRI. Lack of dietary diversity in Ethiopia is tied to limited access to a variety of foods and affordability, new research finds [Internet]. [cited 2022 Apr 11]. Available from: http://www.aliveandthrive.org/en/news/lack-of-dietary-diversity-in-ethiopia-is-tied-to-limited-access-to-a-variety-of-foods-and.

[34] Bai Y, Alemu R, Block SA, Headey D, Masters WA (2021). Cost and affordability of nutritious diets at retail prices: evidence from 177 countries. Food Policy.

[35] Hirvonen K, Bai Y, Headey D, Masters WA (2020). Affordability of the EAT-Lancet reference diet: a global analysis. Lancet Glob Health.

[36] Bai Y, Herforth A, Masters WA (2022). Global variation in the cost of a nutrient-adequate diet by population group: an observational study. Lancet Planet Health.

[37] Berhane HY, Ekström EC, Jirström M, Berhane Y, Turner C, Alsanius BW (2018). What influences urban mothers’ decisions on what to feed their children aged under five—the case of Addis Ababa, Ethiopia. Nutrients.

[38] Trübswasser U, Baye K, Holdsworth M, Loeffen M, Feskens EJ, Talsma EF. Assessing factors influencing adolescents’ dietary behaviours in urban Ethiopia using participatory photography. Public Health Nutr 24(12):3615–23. 10.1017/S1368980020002487 PMC8369459 32792020

[39] Turner C, Aggarwal A, Walls H, Herforth A, Drewnowski A, Coates J (2018). Concepts and critical perspectives for food environment research: a global framework with implications for action in low- and middle-income countries. Glob Food Secur.

[40] Nguyen PH, Avula R, Ruel MT, Saha KK, Ali D, Tran LM (2013). Maternal and Child Dietary Diversity Are Associated in Bangladesh, Vietnam, and Ethiopia. J Nutr.

[41] World Medical Association Declaration of Helsinki (2013). Ethical principles for Medical Research Involving human subjects. JAMA.

