# Supplementary material for: Perception of affordable diet is associated with pre-school children’s diet diversity in Addis Ababa, Ethiopia: the EAT Addis survey
Source: BMC Nutr. 2024 Mar 6;10:47. doi: 10.1186/s40795-024-00859-5 (PMC10916157; doi:10.1186/s40795-024-00859-5)
Supplement: Supplementary file 1 — Supplementary Material 1 [file 40795_2024_859_MOESM1_ESM.docx]

**Perception of affordable diet is associated with pre-school children’s diet diversity in Addis Ababa, Ethiopia: the EAT Addis survey**

Semira Abdelmenan

**Supplemental Figure 1:** Flowchart of households and children included in the Perception of affordable diet is associated with pre-school children’s diet diversity in Addis Ababa, Ethiopia: the EAT Addis survey

**Supplemental Table 1:** Mixed effect linear regression model results

| Variables | | Model 1 | Model 2 | Model 3 |
| --- | --- | --- | --- | --- |
| Affordability of DD | | 0.23^*^ [0.21,0.25] | 0.18^*^[0.16,0.20] | 0.17^*^[0.15,0.19] |
| Education | No education | ref | ref | ref |
|  | Grade 1-4 | 0.00[-0.16,0.17] | -0.04[-0.20,0.12] | -0.03[-0.19,0.13] |
|  | Grade 5-8 | 0.30^*^[0.17,0.42] | 0.12^+^[-0.00,0.25] | 0.13^*^[0.01,0.26] |
|  | Grade 9-12 | 0.68^*^[0.55,0.81] | 0.34^*^[0.22,0.47] | 0.34^*^[0.21,0.48] |
|  | College | 0.94^*^[0.80,1.08] | 0.46^*^[0.32,0.60] | 0.46^*^[0.32,0.61] |
| Wealth status | lowest | ref | ref | ref |
|  | second | 0.34^*^[0.22,0.46] | 0.20^*^[0.08,0.32] | 0.20^*^[0.08,0.32] |
|  | middle | 0.54^*^[0.41,0.66] | 0.28^*^[0.16,0.41] | 0.27^*^[0.15,0.39] |
|  | fourth | 0.56^*^[0.44,0.69] | 0.29^*^[0.17,0.41] | 0.28^*^[0.15,0.40] |
|  | highest | 0.99^*^[0.87,1.12] | 0.53^*^[0.40,0.65] | 0.51^*^[0.38,0.64] |

95% confidence intervals in brackets

^+^ *p* < 0.10, ^*^ *p* < 0.05

Model 1 is bivariate (unadjusted) models. Model 2 is adjusted for affordability of food, maternal education and wealth status. Model 3 is adjusted for affordability of food, maternal education, wealth status, maternal age, family size, seasonal variation, and household food insecurity. All models accounted for the clustering in the data.
